# Loss of RhoB Expression Enhances the Myelodysplastic Phenotype of Mammalian Diaphanous-Related Formin mDia1 Knockout Mice

**DOI:** 10.1371/journal.pone.0007102

**Published:** 2009-09-21

**Authors:** Aaron D. DeWard, Kellie Leali, Richard A. West, George C. Prendergast, Arthur S. Alberts

**Affiliations:** 1 Laboratory of Cell Structure and Signal Integration, Van Andel Research Institute, Grand Rapids, Michigan, United States of America; 2 Flow Cytometry Core Facility, Van Andel Research Institute, Grand Rapids, Michigan, United States of America; 3 Program in Cell and Molecular Biology, Michigan State University, East Lansing, Michigan, United States of America; 4 Lankenau Institute for Medical Research, Wynnewood, Pennsylvania, United States of America; Roswell Park Cancer Institute, United States of America

## Abstract

Myelodysplastic syndrome (MDS) is characterized by ineffective hematopoiesis and hyperplastic bone marrow. Complete loss or interstitial deletions of the long arm of chromosome 5 occur frequently in MDS. One candidate tumor suppressor on 5q is the mammalian Diaphanous (mDia)-related formin mDia1, encoded by *DIAPH1* (5q31.3). mDia-family formins act as effectors for Rho-family small GTP-binding proteins including RhoB, which has also been shown to possess tumor suppressor activity. Mice lacking the *Drf1* gene that encodes mDia1 develop age-dependent myelodysplastic features. We crossed mDia1 and RhoB knockout mice to test whether the additional loss of RhoB expression would compound the myelodysplastic phenotype. *Drf1* ^−/−^ *RhoB* ^−/−^ mice are fertile and develop normally. Relative to age-matched *Drf1* ^−/−^ *RhoB* ^+/−^ mice, the age of myelodysplasia onset was earlier in *Drf1* ^−/−^ *RhoB* ^−/−^ animals—including abnormally shaped erythrocytes, splenomegaly, and extramedullary hematopoiesis. In addition, we observed a statistically significant increase in the number of activated monocytes/macrophages in both the spleen and bone marrow of *Drf1* ^−/−^ *RhoB* ^−/−^ mice relative to *Drf1* ^−/−^ *RhoB* ^+/−^ mice. These data suggest a role for RhoB-regulated mDia1 in the regulation of hematopoietic progenitor cells.

## Introduction

mDia-family formins assemble linear actin filaments and modulate microtubule dynamics in response to adhesive and proliferative stimuli [1]. They are regulated by Rho-family small GTP-binding proteins such as RhoB [2], [3]. Rho GTPases activate formins through direct binding and disruption of an autoinhibitory mechanism mediated by regulatory domains that flank the actin/microtubule-binding formin homology-2 (FH2) domain [1]. While the roles of mDia formins in directed cell migration, cell division, and development are well established [4], only recently have gene-targeting experiments in mice revealed roles for mDia family formins in immune and myeloid cell proliferation [5], [6], [7].

The *DIAPH1* gene encoding human mDia1 (located at 5q31.3) lies between the two commonly deleted regions mapped by conventional cytogenetics in myelodysplastic syndrome (MDS) patient samples [8]. 5q- MDS is characterized by peripheral cytopenias and ineffective hematopoiesis [9]. How defects in one or more 5q genes trigger MDS or contribute to malignant progression in conjunction with additional chromosomal abnormalities remains unknown [10]. Gene targeting experiments on the murine *DIAPH1* homolog *Drf1* show that loss of mDia1 expression impairs the growth control of hematopoietic progenitors [6]. However, the specific mechanism by which loss of mDia1 expression triggers the MDS-like phenotype is currently under investigation.

The small GTPase RhoB binds to both mDia1 and mDia2 on endosomes and has a role in endosome trafficking [2], [3]. RhoB also plays an important role in the apoptotic response to DNA damage [11], and loss of RhoB expression has been shown to correlate with late-stage malignancy [12], [13]. Mice lacking RhoB alone do not show any signs of myelodysplasia or any developmental or fertility defects, but Ras-transformed mouse embryonic fibroblasts (MEFs) from these mice are resistant to apoptosis in the presence of farnesyltransferase inhibitors (FTIs), doxorubicin, or taxol [14], [15], [16]. Together, these studies suggest RhoB possesses tumor suppressor activity.

In this study, we hypothesized that the myelodysplasia observed in *Drf1*-null mice would be enhanced by the additional loss of one of its regulators, RhoB. After examination of the peripheral blood, bone marrow, and spleen hematopoietic progenitor cells, we show that *Drf1* ^−/−^ *RhoB* ^−/−^ mice develop age-dependent myelodysplasia before *Drf1* ^−/−^ *RhoB* ^+/−^ mice. These results are consistent with a model for disease progression in MDS that includes the alteration of multiple tumor suppressors in hematopoietic stem or progenitor cells.

## Materials and Methods

### Gene targeting

The gene targeting and genotyping of *Drf1*-null mice were exactly as previously described [17]; targeting and genotyping of *RhoB*-null mice were as described in [15]. The mice used in this study were a mixed 129/B6 genetic background. For all experiments, data was acquired from six *Drf1* ^−/−^ *RhoB* ^+/−^ and six *Drf1* ^−/−^ *RhoB* ^−/−^ mice at 100 days of age, and from eight *Drf1* ^−/−^ *RhoB* ^+/−^ and nine *Drf1* ^−/−^ *RhoB* ^−/−^ mice at 400 days of age.

### Ethics Statement

All experiments performed were approved in advance by the Van Andel Research Institute Institutional Animal Care and Use Committee.

### Flow cytometry analysis

Peripheral blood, bone marrow, and splenic single-cell suspensions were characterized by flow cytometric analysis. Peripheral blood was extracted from the heart using a syringe equipped with a fine gauge needle. Bone marrow was flushed from femurs using a syringe with a fine gauge needle and 3 mL of PBS. Single-cell suspensions of the spleen were obtained by mincing tissue with glass slides and subsequent passage and scraping of tissue in a ThermoShandon biopsy bag (Thermo Fisher Scientific).

Cells were incubated for 15 min at 20°C in the dark. Incubation was followed by addition of 1× FACSLyse reagent (Becton Dickinson) for 15 min at 20°C in the dark. After RBC lysis, the remaining cells were washed in 2 mL PBS with 0.1% sodium azide. Cells were fixed in 1.0% methanol-free formaldehyde (Polysciences, Inc.) in PBS containing 0.1% bovine serum albumin and refrigerated at 4.0°C until acquisition. Appropriate subclass and negative controls were used to detect nonspecific binding of antibody and autofluorescence. A minimum of 10,000 events for fresh mononuclear cells and 5,000 events for splenic cells were acquired. Flow cytometric analyses were conducted using either a FACSCalibur 4-color or a FACSAria 12-color flow cytometer (Becton Dickinson). Data were analyzed using Becton Dickinson CellQuest Pro and FACSDiVa software.

### Monoclonal antibodies

The following monoclonal antibodies were used: anti-CD29APC from BioLegend; anti-CD45PerCP (30-F11), anti-CD41FITC (MWReg30), anti-CD71FITC (C2), anti-CD74FITC (In-1), anti-TER-119PE (Ly-76, Ter-119), anti-CD13PE (R3-242), anti-CD19PE (1D3), and anti-CD11bAPC (D12) from BD PharMingen; and anti-F4/80 (BM8), anti-CD8aPE (5H10), anti-CD4APC (RM4-5), anti-CD34APC (MEC14.7), and anti-CD3FITC (500A2) from Invitrogen/Caltag Laboratories.

### Cell cycle analysis, complete blood count (CBC), and statistics

Cell cycle analyses used propidium iodide (Sigma) in a modified Vindelov's preparation. A minimum of 10,000 events were collected by flow cytometry. Data were analyzed using Becton Dickinson CellQUEST Pro and Verity House ModFIT LT software. CBC analysis was performed using a VetScan HM2 Hematology System (Abaxis). All statistical analysis was performed using the one-tailed Mann Whitney test for significance. Each point on scatter plots represent a single mouse, and each plot includes a horizontal line to indicate the median of the data. Scatter plots and statistics were performed using GraphPad Prism 5.0 software.

## Results

We previously reported that *Drf1* ^−/−^ and *Drf1* ^+/−^ mice develop age-dependent myelodysplasia, typically around 450 days of age [6]. We asked whether the additional homozygous loss of *RhoB* would enhance disease progression relative to *Drf1*-null mice that still contain a functional allele of *RhoB*. Previous reports have shown that *RhoB* ^+/−^ cells are indistinguishable from *RhoB* ^+/+^ cells in their apoptotic response to DNA damage [16]. We characterized multiple mice of each genotype at 100 and 400 days of age to assess the presence of myelodysplastic features.

**Figure 1:**
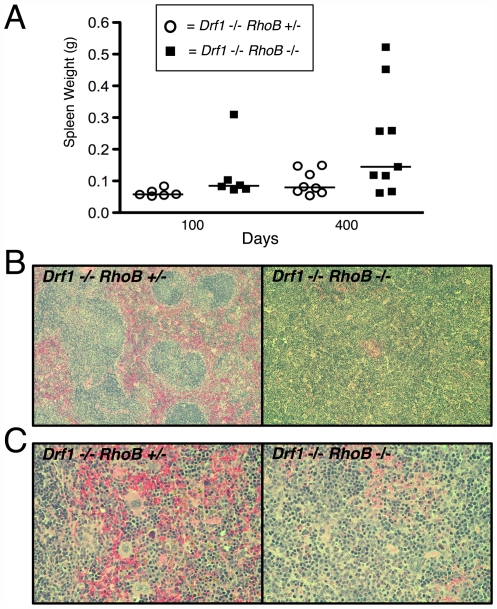
Mice lacking *Drf1* and *RhoB* develop splenomegaly and splenic disorganization. *Drf1* ^−/−^ *RhoB* ^−/−^ and *Drf1* ^−/−^ *RhoB* ^+/−^ mice were necropsied at 100 and 400 days of age. *A*. Spleens were removed from mice and weighed immediately. Each point on scatter plot represents data from a single mouse. *B*. Formalin-fixed spleens from 400-day-old mice were paraffin-embedded and stained with H&E. Sections shown are at 10×magnification. *C*. Splenic sections from *B*, but at 40×magnification.

Upon necropsy, we found that several 400-day-old *Drf1* ^−/−^ *RhoB* ^−/−^ mice had splenomegaly, as determined by whole spleen weight compared to that of *Drf1* ^−/−^ *RhoB* ^+/−^ mice (Fig. 1*A*). Pathological examination of H&E-stained splenic sections showed significant dysplasia, and there was often atrophy of the white pulp with poorly formed germinal centers (Fig. 1*B*). Splenic sections from *Drf1* ^−/−^ *RhoB* ^−/−^ mice also revealed abnormal ratios of myeloid and erythroid composition (frequently a myeloid:erythroid ratio greater than 2.0) and an increased presence of extramedullary hematopoiesis (EMH) (Fig. 1*C*).

**Figure 2:**
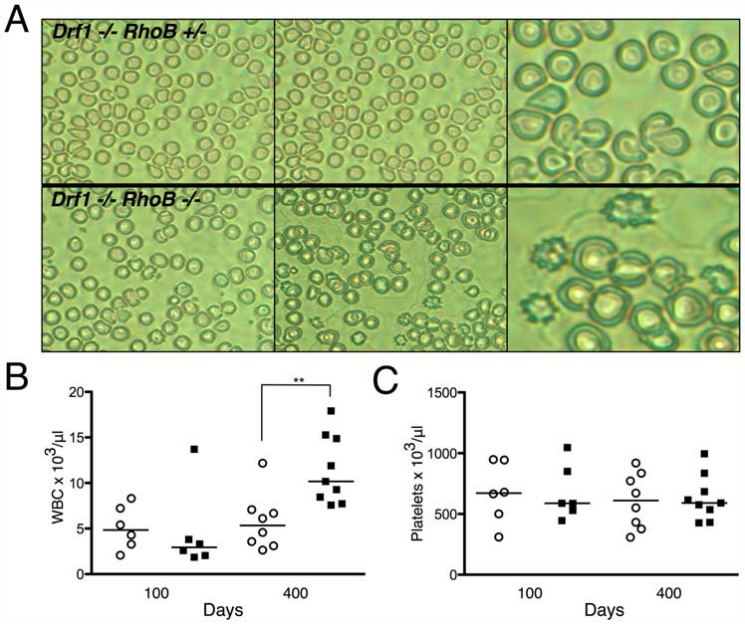
Peripheral blood from *Drf1* ^−/−^ *RhoB* ^−/−^ mice show age-dependent abnormalities. *A*. Peripheral blood smears stained with Wright-Giemsa from 400-day-old mice. Left and center panels are images at 40x; right panels are 60x. *B*. Total WBC count from peripheral blood. *C*. Platelet numbers from peripheral blood CBC analysis. In both *B* and *C*, each point on scatter plot represents data from a single mouse (o = *Drf1* ^−/−^ *RhoB* ^+/−^; ▪ = *Drf1* ^−/−^ *RhoB* ^−/−^) (** denotes *P*≤0.01).

Based on our previous work in *Drf1*-null mice [6] and the recent finding that the mDia2 formin contributes to erythroblast enucleation [18], we examined the peripheral blood to determine if there was morphological evidence of erythrocyte dysplasia. Peripheral blood smears from *Drf1* ^−/−^ *RhoB* ^−/−^ mice showed a marked elevation in abnormally shaped erythrocytes compared with blood from *Drf1* ^−/−^ *RhoB* ^+/−^ mice. The erythrocytes were often spiked (echinocyte) or teardrop in appearance (Fig. 2*A*), consistent with dysplastic features observed in patients with MDS. *Drf1* ^−/−^ *RhoB* ^+/−^ mice did show signs of dysplasia at 400 days, with several teardrop-shaped erythrocytes, but to a lesser extent than *Drf1* ^−/−^ *RhoB* ^−/−^ mice. CBC analysis of peripheral blood revealed a significant increase in the WBC count of *Drf1* ^−/−^ *RhoB* ^−/−^ mice relative to that of *Drf1* ^−/−^ *RhoB* ^+/−^ mice at 400 days of age (Fig. 2*B*). Abnormal platelet counts are sometimes observed in certain subsets of MDS, but we did not observe any significant differences in these mice (Fig. 2*C*).

**Figure 3:**
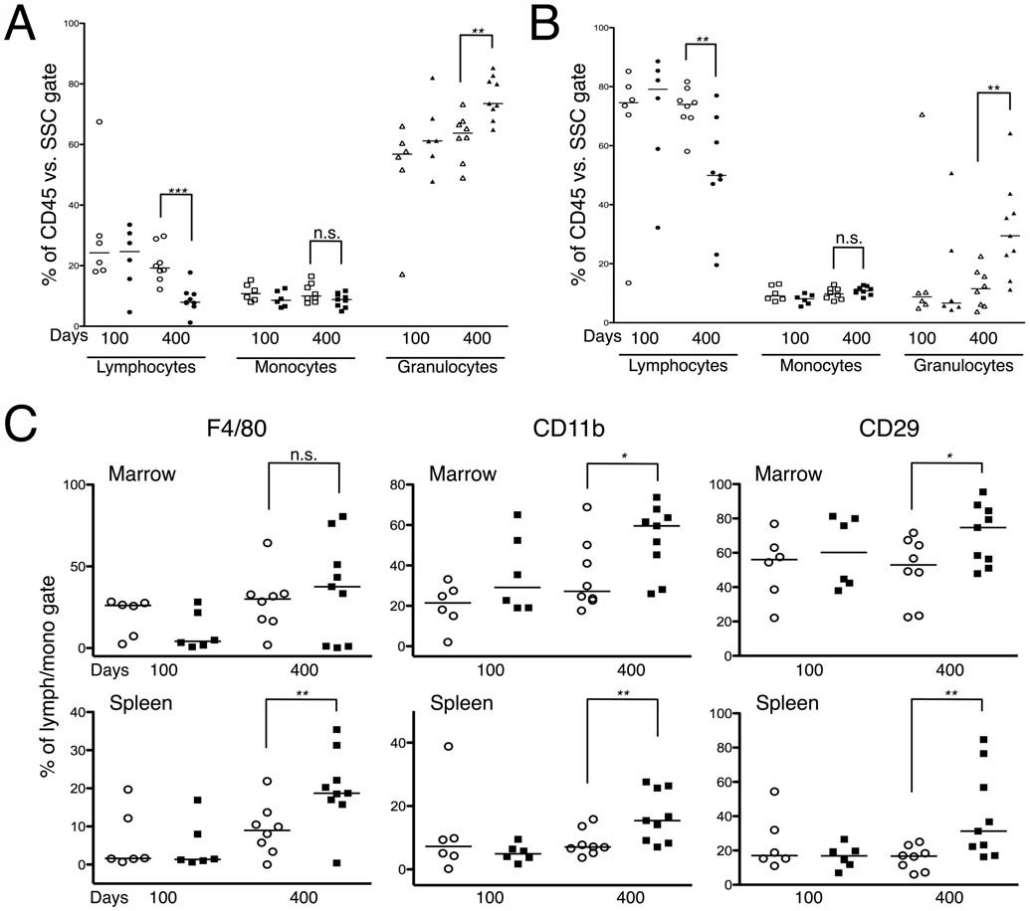
Flow cytometry analysis of mouse bone marrow and splenic cells. *A*. Scatter plot showing the percentage of lymphocytes, monocytes, and granulocytes from bone morrow. Open shapes represent *Drf1* ^−/−^ *RhoB* ^+/−^ mice and filled shapes represent *Drf1* ^−/−^ *RhoB* ^−/−^ mice (** denotes *P*≤0.01; *** denotes *P*≤0.001). *B*. Scatter plot showing the percentage of lymphocytes, monocytes, and granulocytes from mice splenic single-cell suspensions. Legend is the same as in *A* (** denotes *P*≤0.01). *C*. Percentage of F4/80^+^, CD11b^+^, and CD29^+^ cells from the bone marrow and spleen of mice (* denotes *P*≤0.05; ** denotes *P*≤0.01) (o = *Drf1* ^−/−^ *RhoB* ^+/−^; ▪ = *Drf1* ^−/−^ *RhoB* ^−/−^).

We then focused our analysis specifically on the bone marrow and spleen to examine myelopoiesis in these compartments. Flow cytometry was used to determine the percentage of lymphocytes, monocytes, and granulocytes based on the pan-leukocyte marker CD45. Using the gating strategy outlined previously [6], we found that 400-day-old *Drf1* ^−/−^ *RhoB* ^−/−^ mice had an increased percentage of granulocytes and a concomitant decrease in lymphocytes in the bone marrow compartment (Fig. 3*A*). Analysis of splenic single-cell suspensions isolated from 400-day-old mice showed a similar increase of granulocytes in *Drf1* ^−/−^ *RhoB* ^−/−^ mice (Fig. 3*B*).

To further examine potential differences in myelopoiesis between *Drf1* ^−/−^ *RhoB* ^+/−^ mice and *Drf1* ^−/−^ *RhoB* ^−/−^ mice, we performed flow cytometry on cells from the bone marrow and spleen to detect levels of F4/80 (a pan macrophage marker) and CD11b (integrin αM; monocyte development marker). By 400 days of age, *Drf1* ^−/−^ *RhoB* ^−/−^ mice had an increased percentage of F4/80^+^ cells in the spleen, but we observed no significant difference in the bone marrow (Fig. 3*C*). On the other hand, CD11b^+^ cells were significantly elevated in both the marrow and spleen by 400 days of age. We also examined cellular expression of the marker CD29 (β1 integrin; important for homing and retention in lymphoid organs). We found an increased percentage of CD29^+^ cells in the marrow, with an even more pronounced increase in the spleen of *Drf1* ^−/−^ *RhoB* ^−/−^ mice relative to *Drf1* ^−/−^ *RhoB* ^+/−^ mice (Fig. 3*C*). These results are consistent with the observed increase in myeloid cell proportion found by histopathology (Fig. 1).

**Figure 4:**
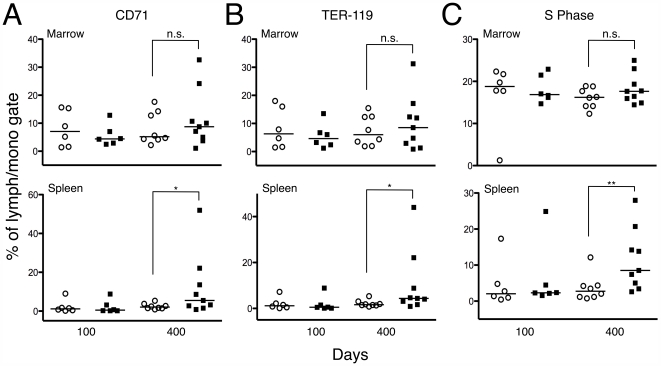
Flow cytometry analysis of erythroid precursors in mouse bone marrow and spleen. Each point on the scatter plots represents data from a single mouse (o = *Drf1* ^−/−^ *RhoB* ^+/−^; ▪ = *Drf1* ^−/−^ *RhoB* ^−/−^) *A*. Scatter plot showing the percentage of CD71^+^ cells from the bone marrow and spleen of mice (* denotes *P*≤0.05). *B*. Percentage of TER-119+ cells from the bone marrow and spleen of mice (* denotes *P*≤0.05). *C*. Percentage of cells undergoing S phase from the bone marrow and spleen of mice (** denotes *P*≤0.01).

Finally, we analyzed the levels of TER-119 (erythroid-specific marker) and CD71 (transferrin receptor; a marker of proliferating erythroid precursors) in the marrow and spleen of *Drf1* ^−/−^ *RhoB* ^−/−^ and *Drf1* ^−/−^ *RhoB* ^+/−^ mice. While there were no differences in the bone marrow, the levels of CD71^+^ and TER-119^+^ cells were elevated in the spleens of 400-day-old *Drf1* ^−/−^ *RhoB* ^−/−^ mice (Fig. 4, *A* and *B*). An increase in erythroid precursors was also evident in H&E-stained splenic sections (data not shown). Consistent with this observation and the presence of splenic EMH, we also found a substantial increase in splenic cells undergoing S phase in 400-day-old *Drf1* ^−/−^ *RhoB* ^−/−^ mice relative to the number in *Drf1* ^−/−^ *RhoB* ^+/−^ mice (Fig. 4*C*).

## Discussion

MDS is thought to arise because of multiple alterations in a hematopoietic stem cell [10]. We previously found that knocking out mDia1 expression in mice leads to the age-dependent development of myelodysplasia [6]. Here, we show that the additional knockout of RhoB expression in *Drf1*-null mice accelerates the progression to myelodysplasia. Several candidate tumor suppressor genes in humans reside on the long arm of chromosome 5 [8]. One of these genes is *DIAPH1*, which encodes the actin assembly protein mDia1. While genetic ablation of mDia1 expression alone in mice resembles 5q- MDS, it is likely that mDia1 acts in concert with multiple other genes on the same commonly deleted region to suppress malignancy [8].

MDS can progress to a more advanced stage and ultimately develop into leukemia. The transformation events involved in disease progression are thought to include genes that contribute to cell-cycle control, apoptosis, and differentiation [10]. The small GTPase RhoB is required for apoptosis in response to DNA-damaging agents and farnesyltransferase inhibitors [14], [16]. Previous reports have shown that RhoB negatively regulates Akt survival signaling and mediates apoptosis in a p53-dependent manner [19], [20]. Given its role in apoptosis and its known interactions with the mDia formins, we hypothesized that deletion of *RhoB* would enhance the progression of MDS in mice lacking mDia1 expression. Our results support this hypothesis and further substantiate our mouse model of age-associated myelodysplasia. It is also interesting to speculate that loss of RhoB expression may interfere with hematopoietic stem cell maintenance, since recent work has highlighted a role for *RhoB* in stem cell self-renewal [21]. Together, these findings suggest that multiple mechanisms may contribute to the myelodysplastic phenotype in our mice.

Our mouse model uniquely positions us to test some important questions related to the *in vivo* role of RhoB in the anti-neoplastic affects of FTIs. FTIs have been tested clinically to treat various myelodysplasias, with varying degrees of efficacy [22]. But whether FTIs can alleviate the disease phenotype in *Drf1*-null or heterozygous mice and whether RhoB is required *in vivo* to mediate this response remains to be determined.

Our results point to the down-regulation of RhoB as a potential marker for late-stage MDS, similar to its diminished expression in other late-stage cancers [12], [13]. Examination of the gene expression profile of RhoB in human patient samples that have a more advanced disease signature could help determine better treatment options for patients diagnosed with MDS.

In summary, we show that mice lacking mDia1 and RhoB expression progress to MDS faster than mice lacking mDia1 alone. These data parallel observations of MDS in humans, in which multiple alterations in hematopoietic stem cells contribute to disease pathogenesis. Our mouse model will be useful in characterizing the mechanism of disease progression further and in testing potential therapeutics to treat this chronic disease.
